# Toward a Common Set of Interface Requirements for Genomic Data Management: Scoping Review

**DOI:** 10.2196/78405

**Published:** 2026-04-27

**Authors:** Valeria Resendez, Funda Yıldırım, Eugenio Gaeta, Giuseppe Fico, Simone Borsci

**Affiliations:** 1 University of Twente Enschede The Netherlands; 2 Universidad Politécnica de Madrid Madrid Spain; 3 Imperial College London London United Kingdom

**Keywords:** functional requirements, nonfunctional requirements, genomics, electronic health records, interfaces, data sharing

## Abstract

**Background:**

Genomic data can advance precision medicine; however, to continue developing more targeted treatments, genomic datasets need to be integrated with health care data and become more disease-focused. This integration, in turn, amplifies existing challenges in health care data management, such as handling large data volumes, adhering to data standards, and protecting sensitive information. Addressing these challenges calls for unified digital ecosystems that combine data collection, standardization, analysis, and governance within a single platform, thereby reducing the technical burden for users. Currently, a clear set of indications about functional and nonfunctional requirements to help designers translate stakeholder needs into actionable design specifications is missing.

**Objective:**

This scoping review aimed to identify the functional and nonfunctional requirements most frequently discussed in the literature from the perspective of end users (eg, clinicians and data analysts) to inform the design of a health and genomic data management platform that supports data sharing and analysis in clinical settings by conducting a PRISMA-ScR (Preferred Reporting Items for Systematic reviews and Meta-Analyses extension for Scoping Review) review.

**Methods:**

We searched for peer-reviewed English studies that focused on platforms for managing genomic data from a user-centered perspective. We considered studies from 2014 to 2024 that were extracted from Scopus, PubMed, Web of Science, and Google Scholar for the scoping review. Insights were extrapolated for a thematic analysis to develop an initial set of requirements. We charted the functional and nonfunctional requirements according to their frequency of occurrence in the literature to provide a structured overview of the most commonly reported requirements.

**Results:**

From 410 initial items, 210 items were preliminarily selected, and 53 items were included in the final analysis. Three primary groups of 26 interface functional requirements emerged: (1) general data management (acquisition, standardization, and sharing), (2) data processing and analysis (preprocessing and analysis pipelines), and (3) data visualization and reporting. Twenty nonfunctional requirements were identified and organized in 4 groups: (1) communication and support, (2) platform technical infrastructure, (3) user experience and user interface characteristics, and (4) security and compliance. We also investigated the issues that need to be resolved to develop an ideal platform.

**Conclusions:**

We identified and mapped the most frequently reported functional and nonfunctional requirements of clinical and data professionals when discussing a health and genomic data management platform. The 3 key functional requirements should be supported by nonfunctional requirements such as secure technical infrastructure and governance mechanisms that enable compliant data processing and sharing. Designers may use these insights and mapping to develop standardized data platforms that promote efficient data exchange between institutions and experts while ensuring regulatory compliance and secure access, as proposed by the European Health Data Space.

## Introduction

### Background

Since the completion of the Human Genome Project in 2003, which marked a turning point in genomic research, advancements in sequencing technologies have increased both the volume and complexity of genomic data [1,2]. For nearly 2 decades, there has been a continuous evolution in genomic data management systems and the analytical methods used in genomic research. Genomic data play a vital role in enhancing patient treatments, from pharmaceutical developments [3] to improving outcomes of organ transplants [4]. Currently, many genomic and health care datasets are isolated or not disease-focused [5]. This certainly limits clinicians' and researchers' ability to advance precision medicine and develop more targeted treatments [6]. However, when health care data are combined with genomic data, the interoperability, computational, and legal challenges increase in complexity due to the quantity and size of data and to the stricter processing requirements. As Alzu’bi et al [1] highlight, platforms designed to manage, share, and analyze genomic data must tackle at least four challenges, which are (1) handle overwhelming volumes of data that often require preprocessing and standardization, especially important for genomic data; (2) simplify complex analysis for highly specialized (large set of) genomic data [1,4]; (3) manage data in line with the legal, social, and ethical issues associated with personal genomic information (eg, preprocessing and analyzing genomic data requires safeguarding patient privacy in line with national and international regulatory frameworks, as well as control to determine who is authorized to access the data [1,4]); and (4) ensure the security and privacy of genomic data through regulatory compliance. This means incorporating regulatory principles such as the General Data Protection Regulation (GDPR) [7], the European Data Act [8], and other European Regulations from the earliest stages of design. Both the platform and its intended use should be developed to maximize data protection and to promote secure data handling practices by design.

The latter challenge (ie, ensuring the security and privacy of the data) aligns with broader European initiatives aimed at creating a framework for health data management and exchange. For instance, the European Health Data Space [9] represents a key pillar of the European health global strategy [10]. The European Health Data Space is the ecosystem of rules, standards, practices, and infrastructures that aims to address the ethical and technical challenges of exchanging interoperable health data in a very diverse and complex environment like the European Union. Interoperability in this context refers to the ability of organizations to work collaboratively toward shared goals by exchanging information and knowledge [11]. The European context is not the only one that deals with the issues of ensuring the privacy and security of systems for exchanging health data. Recently, the US Department of Health and Human Services published an update on the US health care privacy rule [12]. How the EU and US companies, as well as practitioners in both systems, will access and exchange data with reciprocal advantages is the potential point of connection among the regulatory frameworks currently under discussion [13].

Enabling well-regulated ecosystems to exchange data (to a certain extent and in line with regulations) is an essential aspect of supporting responsible research, fostering innovation, informing policymaking, enhancing patient safety, and streamlining regulatory activities [14]. For the specific case of genomics, global initiatives, such as the Global Alliance for Genomics and Health (GA4GH) [15], ELIXIR [16], and the Genomic Data Infrastructure (GDI) [17], are working to tackle the challenges described above by promoting standards for genomic data sharing. For instance, GA4GH focuses on creating a common framework that enables effective and responsible data sharing, driving progress in genomic research and medicine. ELIXIR works to coordinate infrastructural resources, such as databases, software tools, training materials, cloud storage, and supercomputers, facilitating data discovery, expertise exchange, and the establishment of best practices. Meanwhile, GDI strives to provide access to genomic, phenotypic, and clinical data across Europe by establishing a federated, sustainable, and secure infrastructure for data access. Similarly, the European Union Joint Action Towards a European Health Data Space (TEHDAS) program reflects the broader European move toward a federated approach to health data exchange, including genomic information. Under a federated approach, data remains locally stored but can be queried and analyzed across sites [18]. Together, these initiatives illustrate an international recognition that responsible data sharing is fundamental to advancing health and genomic research and realizing the potential of precision medicine [19]. At the same time, the initiatives show a shift toward infrastructures that are interoperable, secure, and designed for collaboration at scale, ensuring that sensitive data can be used to generate scientific and clinical insights while respecting legal and ethical constraints.

A key challenge in improving health and genomic data management, as well as facilitating data exchange in a federated modality, is developing digital ecosystems that can unify data collection, standardization, exchange, and analysis on a single platform. By integrating data access, governance controls, preprocessing tools, analytical workflows, and visualization capabilities into a single interface platform, the main advantage will be providing clinical experts with access to the same datasets. This will also harmonize the analytical power and possibilities of analysis for operators in different countries [20,21]. These types of platforms can also support regulatory compliance and broaden participation by making genomic analysis accessible beyond bioinformaticians [2,22]. Enabling the accessibility of such information to different stakeholders is key to further advancing precision medicine and allowing more personalized treatments [23].

The development of a unifying federated digital ecosystem (ie, unified front-end with distributed data) needs the definition of requirements. Requirements specify the services a system must provide and the constraints under which it must operate [24]. Describing requirements through a categorization into functional and nonfunctional enables the first high-level translation of needs into specifications that (with further refinements) can be used by designers and developers to build a platform. In this work, we build on previous definitions and define such requirements as follows:

1. Functional: These requirements refer to the platform’s ability to provide operations that serve (stated or implied) the needs of the end users [25]. These functional requirements represent what the system can and must do [24,26].
2. Nonfunctional: These requirements are qualities of the platform that support the different functionalities to be used appropriately (eg, accessibility and usability) [27]. These nonfunctional requirements also define the constraints of the software architecture [24,26,28]. Nonfunctional requirements often apply to the system as a whole rather than individual system features or services (eg, security, access control, and availability).

Functional and nonfunctional requirements are entangled aspects; for example, empowering nontechnical users to analyze data easily could involve functional requirements like drag-and-drop interfaces or prebuilt analysis templates, and is connected to nonfunctional requirements such as accessibility and perceived usability [2,29-31].

### Previous Work on Genomics Platform Requirements

Several projects have been in the past (and are currently) investigating and developing functional and nonfunctional requirements to enable, for instance, more secure exchanges of health and genomic data. For example, the IntelliOmics project is developing a distributed system that anonymizes data before making it accessible to users [4]. Similarly, the Integrated Microbial Genomes platform offers an interface with privacy functional requirements that allow users to decide whether they want their annotations and data to be public or private [32], as well as the Beacon v2 platform of GDI that provides data discovery functionalities designed to facilitate the search for genomic variants and associated information across distributed datasets without compromising data privacy [33,34].

St. Jude Cloud software (St. Jude Children’s Research Hospital) provides a facilitated process of data integration by standardizing data collection and management through different steps of the data analysis process [2]. Additionally, this system uses open-source tools and common data standards to make it easier for researchers to share and combine diverse datasets for in-depth analysis [2]. In a similar way, to simplify data processing, the MetaboAnalyst initiative developed a system with a set of functionalities that allow for easier data preprocessing while providing interactive exploration for users [31]. Another example is the open platform BinaRena, which aims to enhance data visualization by rendering interactive scatter plots that integrate multiple data layers, thereby facilitating the delineation of microbial communities even in extensive datasets [35]. These projects collectively address key challenges in genomic data management. Nevertheless, we are not aware of any structured and systematic investigation that has mapped and listed a set of the most relevant functional and nonfunctional requirements for the potential end users and stakeholders of health and genomic data management.

A systematic and in-depth analysis of the functional and nonfunctional requirements reported in literature can provide a baseline set of requirements that can be used for future investigations with different stakeholders (ie, clinicians, developers, and statisticians). This analysis can also facilitate the development of genomic and health data platforms that are recognized for their levels of perceived quality in usage, such as usability [36] and user experience (UX; ISO 9241-210 [37]). Mapping functional and nonfunctional requirements is the first step in this process. It translates user needs, workflows, and system qualities into design characteristics that specify what the interface must offer. This indicates possible directions for the development of the system. These design characteristics align with the human-centric usability engineering process for medical [38] and software systems [39]. This process can result in the development of effective, useful, interoperable platforms for different stakeholders, thus maximizing the possibility of uncovering insights that can contribute to more targeted diagnoses and treatments, and improved health outcomes.

### Aim of This Work

To our knowledge, no previous work has systematically mapped literature in order to identify a set of functional and nonfunctional requirements for genomic data management platforms commonly identified as important by experts. To address this gap, we conducted a scoping literature review following the PRISMA-ScR (Preferred Reporting Items for Systematic reviews and Meta-Analyses extension for Scoping Reviews) guidelines [39], which is usually adopted to explore a given field and establish standard practices and requirements, for example, study by Piškur et al [40]. Our research question aimed to identify the key requirements (functional and nonfunctional) considered important by experts and should be incorporated into platforms for genomic and health care data management.

This review provides 2 main contributions. First, it translates scattered insights from the literature into a structured set of functional and nonfunctional requirements, which can inform the development of a unified federated ecosystem. Second, it quantifies these requirements to identify design priorities and areas needing further attention. These contributions are particularly relevant for developers and designers of health care tools seeking to improve genomic research through safer, usable, accessible, and acceptable platforms.

## Methods

### Overview

We conducted a scoping review of platforms managing genomic data and reported our findings according to the PRISMA-ScR (Multimedia Appendix 1) [41]. The project was registered retrospectively under the open science framework [42].

### Information Sources and Search Strategy

We conducted a scoping review to map the relevant requirements of platforms managing genomic data. Data searches were conducted between August 9 and August 14, 2024, in Scopus, PubMed, Web of Science, and Google Scholar, searching for peer-reviewed publications published in the last 10 years. The search strategy was organized around three core concepts, (1) data-related practices, (2) genomics, and (3) user-centered or human-centered approaches. Boolean operators (“AND” and “OR”), truncation, and phrase searching were applied to capture variations in terminology.

The inclusion of user-centered and human-centered terms reflects the goal to focus on the identification of user requirements, instead of focusing on features, technical limitations, and feasibility from a technical perspective. Specifically, we wanted to take as much as possible the perspective of the potential end users, including, for instance, researchers, clinicians, or data managers who interact with genomic data. Understanding user needs is key to capturing the functional requirements (eg, what the platform must be able to do) without presupposing the technical solutions or implementation details. This approach allowed us to identify user-facing functionalities, for example, data visualization or data analysis workflows, without the need to commit to technical details (eg, programming language, database schema, or cloud architecture).

For this reason, the following search terms were used to filter titles, abstracts, and keywords in Scopus, PubMed, and Web of Science: “data management” OR “data processing” OR “data exploration” OR “data discovery” OR “data sharing” OR “data analysis” OR “data integration” AND “genomic” OR “genes” OR “DNA” AND “user-centered” OR “user centered” OR “human-centered” OR “human centered” OR “user experience” OR “user requirements” OR “usability.”

In Google Scholar, where filtering by specific fields is not possible, we only applied the same search string to retrieve relevant articles. The aggregated search results were exported into Rayyan, a platform developed for literature reviews, where duplicates were removed. The final selection of the search results can be accessed in the Open Science Framework (OSF) repository for this project [42].

### Eligibility Criteria

The authors established inclusion and exclusion criteria through discussions before the review (refer to Multimedia Appendix 2 for summary). We focused on articles describing key functional and nonfunctional requirements for ensuring a high-level UX, as well as those relevant to user-centered design of health and genomic data platforms. We excluded non-English articles, backend-focused and technical papers, inaccessible content, studies not involving human genomic data management or focusing on specific statistical software analyses, and literature reviews lacking platform requirements. We included only items published from August 2014 to 2024.

### Selection of Source of Evidence

Following PRISMA-ScR guidelines, 1 reviewer (VR) initially screened the abstracts for duplicates and relevance. Then, 2 reviewers (VR and SB) independently assessed titles and abstracts, with a third reviewer (FY) resolving any disagreements. We extracted article metadata (citation count, DOI, publication year, keywords, source, and authors) and both functional and nonfunctional platform requirements from selected articles.

### Data Charting Process

One of the authors (VR) identified potential codes based on the features and aspects reported in the articles as key to the experience of the stakeholders with data platforms. In addition, 2 authors (SB and FY) independently reviewed the initial codes, and disagreements were resolved through iterative discussion until consensus was achieved. The agreed-upon codes were then compared across articles and grouped into broader thematic categories, which formed the basis for the functional and nonfunctional requirements. Requirements describing specific actions, operations, or services the platform must perform (such as data upload, search functions, and workflow execution) were classified as functional. Requirements describing system qualities or constraints (such as security, usability, and scalability) were classified as nonfunctional. The final categorization was reviewed and approved by all authors. The frequency with which requirements appeared across articles was recorded as an indicator of attention in the literature, although this measure should be interpreted cautiously, as it may reflect publication patterns rather than user priorities.

### Data Items

The final selection included 53 data items. From each item, we extracted general bibliographic information, such as title, digital object identifier, publication year, authors, and article type, to enable the easy identification of the data items. In addition, following our data charting procedure, we extracted 108 variables capturing both functional requirements (eg, data access, data upload, and data search) and nonfunctional requirements (eg, security, interoperability, scalability, performance, and accessibility). A full list of the selected data items and the complete set of extracted requirements is available in the project’s OSF repository [42].

### Synthesis of the Results

The full texts of the articles were analyzed to identify all the functionalities of the platform and its characteristics. Through an iterative process of data charting and thematic analysis, these coded characteristics were organized into 2 overarching themes—functional and nonfunctional requirements. These themes reflect how platforms operate and what they do. The functional requirements incorporated themes related to general data management, data processing and analysis, and data visualization and reporting. Nonfunctional requirements capture qualities that enable or constrain these functions. These qualities include the platform’s technical infrastructure, user interface (UI) and UX characteristics, security and compliance mechanisms, and communication and support.

### Study Risk of Bias Assessment

Given the nature of the scoping review and its aim to map the requirements of genomic data management platforms, a formal risk of bias assessment tool was not applied. We adopted an iterative refinement of these criteria before data extraction to further ensure rigor and consistency. The risk of bias in the process of inclusion or exclusion was assessed through a collaborative approach. Two reviewers (VR and SB) independently screened the titles and abstracts of all retrieved articles to minimize subjective biases during the selection process. Disagreements between the 2 reviewers were resolved by consulting a third reviewer (FY).

## Results

### Study Selection

**Figure 1:**
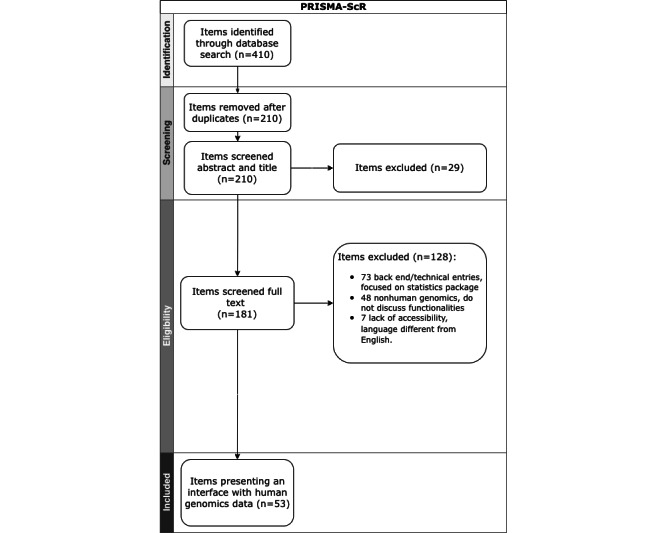
Overview of PRISMA-ScR (Preferred Reporting Items for Systematic reviews and Meta-Analyses extension for Scoping Reviews) procedure.

The PRISMA-ScR flowchart is presented in Figure 1. A total of 410 studies were identified during the database search (151 records from Scopus, 69 from PubMed, 83 from Web of Science, and 107 from Google Scholar). After removing 200 duplicates, the titles and abstracts of the remaining 210 items were screened. After removing 29 items, a total of 181 items were read in full. About 128 items were also excluded. The remaining 53 items met the criteria, that is, providing information regarding essential functional and nonfunctional requirements of platforms related to health and genomic data management.

### Study Characteristics

The items selected for the review include studies investigating features of genomic data management platforms from 2014 to 2024 (peaking during 2019-2022 with 8-9 articles annually), published in a few key journals, for example, “*Nucleic Acids Research*” (8 articles) [31,43-49], “*BMC Bioinformatics*” (5 articles) [50-54], and “*BMC Genomics*” (2 articles) [32,55]. Geographically, the majority of the articles originate from the United States (21 studies) [2,22,29,32,35,44,52,54-67], followed by Canada (8 studies) [31,47,50,68-72], Germany (5 studies) [45,48,53,73-75], Italy (4 studies) [51,53,76,77], the United Kingdom (3 studies) [49,78,79], Belgium (2 studies) [80,81], China (2 studies) [43,82], and 1 article each from Estonia [46], France [83], Greece [84], India [85], Japan [86], Pakistan [87], Poland [4], and Russia [88]. First authors were predominantly male (n=39) versus female (n=14).

### Functional Requirements Groups

#### Overview

**Figure 2:**
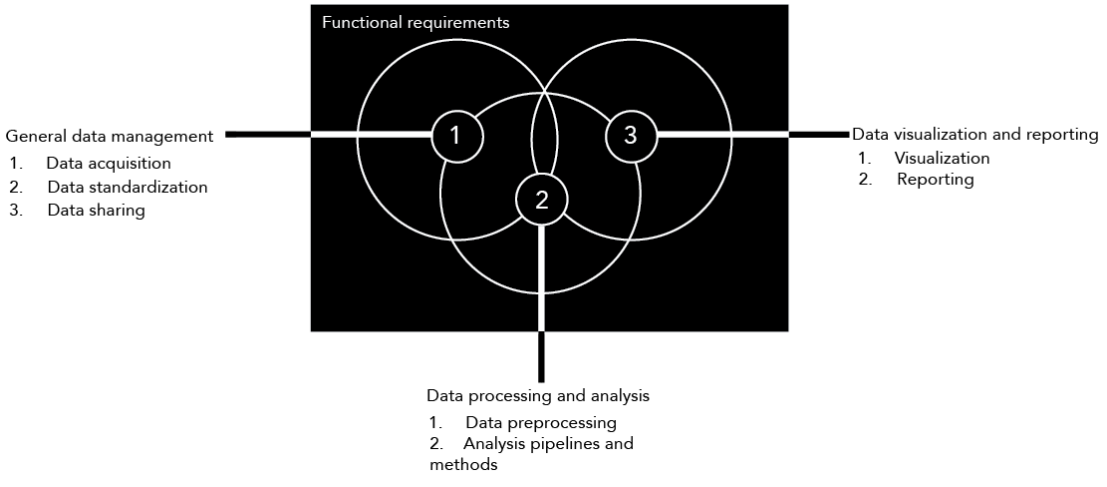
Summary of the key functional requirements for the genomic research process.

We identified 26 functional requirements organized in 3 main groups and associated subgroups (Figure 2) commonly reported in the literature (refer to Multimedia Appendix 3 for full overview). We have described these groups as follows:

1. General data management: This includes essential functional requirements discussed in 98% (n=52) of the articles to ensure appropriate data acquisition, data standardization, and data sharing.
2. Data processing and analysis: This group (discussed in n=53, 100% of the articles) includes key functional requirements for the stakeholders, such as data preprocessing and analysis pipelines and methods.
3. Visualizing data and generating outputs for reporting: This group was discussed in 90% (n=48) of the articles and included the main options for stakeholders to visualize specific types of data and generate reports on the platform.

Associated with the functional requirements, we also mapped the nonfunctional requirements and constraints of the system, that is, inherent characteristics of the platform [24]. Specifically, these requirements include (1) communication and support (39 articles) [2,4,22,31,32,35,43-46,48,53,55-58,60-64,66,68,69, 71,72,74-79,81-87], (2) UX and UI characteristics (50 articles) [2,4,22,29,31,32,35,43-56,58-65,67-85,87,88], (3) security and compliance (32 articles) [2,4,22,29,31,32,48,52-56,58-60, 62-67,70,71,73-75,78,82,84,86-88], and (4) platform technical infrastructure (32 articles) [2,4,22,29,31,46,49,51-54,56-58, 60-67,71,72,74,78,80,82-85]. Table 1 presents an overview of the extent to which various articles address the functional and nonfunctional requirements identified in the analysis.

**Table 1:** Functional and nonfunctional groups of requirements that are mentioned by authors in each selected article.

Study	Main groups of functional requirements				Main group of nonfunctional requirements				Security and compliance
	General data management	Data processing and analysis	Data visualization and reporting	Communication and support		Platform technical infrastructure	UI^a^ and UX^b^ characteristics		
Demchak et al [56]	✓	✓	✓	✓		✓	✓	✓	
Xia et al [68]	✓	✓	✓	✓		—^c^	✓	—	
Sante et al [80]	✓	✓	✓	—		✓	✓	—	
Xia et al [43]	✓	✓	✓	✓		—	✓	—	
Suciu et al [50]	✓	✓	✓	—		—	✓	—	
Sauria et al [57]	✓	✓	—	✓		✓	—	—	
Calabria et al [51]	✓	✓	✓	—		✓	✓	—	
Wolf et al [73]	✓	✓	✓	—		—	✓	✓	
Bhuvaneshwar et al [52]	✓	✓	✓	—		✓	✓	✓	
Murtagh et al [78]	✓	✓	—	✓		✓	✓	✓	
Ding et al [44]	✓	✓	✓	✓		—	✓	—	
Chen I et al [32]	✓	✓	✓	✓		—	✓	✓	
Albuquerque et al [69]	✓	✓	—	✓		—	✓	—	
Davis-Turak et al [29]	✓	✓	✓	—		✓	✓	✓	
Takai-Igarashi et al [86]	✓	✓	✓	✓		—	—	✓	
Danahey et al [58]	✓	✓	✓	✓		✓	✓	✓	
Lau et al [22]	✓	✓	✓	✓		✓	✓	✓	
Crawford et al [59]	✓	✓	✓	—		—	—	✓	
Das et al [70]	✓	✓	✓	—		—	✓	✓	
Warner et al [60]	✓	✓	✓	✓		✓	✓	✓	
Mohr et al [74]	✓	✓	✓	✓		✓	✓	✓	
Pearce et al [61]	✓	✓	✓	✓		✓	✓	—	
Hombach et al [45]	✓	✓	✓	✓		—	✓	—	
Raudvere et al [46]	✓	✓	✓	✓		✓	✓	—	
Nanni et al [53]	✓	✓	✓	✓		✓	✓	✓	
Verbruggen and Menschaert [81]	✓	✓	✓	✓		—	✓	—	
Wünsch et al [75]	✓	✓	✓	✓		—	✓	✓	
Cappelli et al [76]	✓	✓	✓	✓		✓	✓	—	
Kuzmenkov et al [88]	✓	✓	✓	—		—	✓	✓	
Kounelis et al [84]	✓	✓	✓	✓		✓	✓	✓	
Rodchenkov et al [47]	✓	✓	✓	—		—	✓	—	
Bonomi et al [62]	✓	✓	✓	✓		✓	✓	✓	
Yousif et al [54]		✓	✓	—		✓	✓	✓	
Yukselen et al [55]	✓	✓	✓	✓		—	✓	✓	
Holtgrewe et al [48]	✓	✓	✓	✓		—	✓	✓	
Canakoglu et al [77]	✓	✓	✓	✓		—	✓	—	
Ma et al [63]	✓	✓	✓	✓		✓	✓	✓	
Reska et al [4]	✓	✓	✓	✓		✓	✓	✓	
Pang et al [31]	✓	✓	✓	✓		✓	✓	✓	
Campbell et al [64]	✓	✓	✓	✓		✓	✓	✓	
Ochoa et al [49]	✓	✓	✓	—		✓	✓	—	
McLeod et al [2]	✓	✓	✓	✓		✓	✓	✓	
Ullah et al [87]	✓	✓	✓	✓		—	✓	✓	
Raveendran et al [65]	✓	✓	✓	—		✓	✓	✓	
Gilbert et al [79]	✓	✓	✓	✓		—	✓	✓	
Li et al [82]	✓	✓	✓	✓		✓	✓	✓	
Osmond et al [71]	✓	✓	✓	✓		✓	✓	✓	
Reiff et al [66]	✓	✓	✓	✓		✓	✓	—	
Amer-Yahia et al [83]	✓	✓	✓	✓		✓	✓	—	
Pavia et al [35]	✓	✓	✓	✓		—	✓	—	
Post et al [67]	✓	✓	—	—		✓	✓	✓	
Gill et al [72]	✓	✓	✓	✓		✓	✓	—	
Ware et al [85]	✓	✓	—	✓		✓	✓	—	

^a^UI: user interface.

^b^UX: user experience.

^c^Not applicable.

#### General Data Management

General data management encompasses key functionalities of the platform required to enable data analysis and visualization of results [4]. These functionalities can be clustered in three subgroups as described below (Section S1 in Multimedia Appendix 3).

First, data acquisition functionalities refer to the information collected from various sources, including electronic health records (EHRs), laboratory results, open-source databases, and proprietary data from different hospitals [4,83]. These functionalities encompass two main subfunctionalities: (1) the platform’s ability to aggregate data from various sources, including databases, graph structures, and textual sources [83]; and (2) the ability of the platform for users to upload their data [66,72,83,85]. The first subfunctionality is noted in 45 articles [2,4,22,29,31,32,35,45-52,54-59,61-64,66-77,79,80,83-88], and it aims to serve users with abundant information to find undiscovered patterns. The second subfunctionality (ie, allowing users to upload their data) is discussed in 30 articles [2,4,22,29,31,32,44-46,48,50-52,55-57,59,63,68-70,73-76,80,84,85,87,88]. Across the articles, the authors describe a broad range of data formats that users can rely on for upload. These include raw genomic data and genomic variants, such as FASTQ, BAM, and VCF [2,22,29,45,61,80], but also data regarding processed genomic and multiomics, such as CNA, SNP, CpG methylation, mRNA expression, proteomics, or metabolomics profiles [4,61,70], and gene sets or annotation tables including GMT files, CSV, TXT, Excel spreadsheets, BED tracks, and JSON query files [67,72,74]. Several platforms also support uploads of additional auxiliary data types, such as images (eg, PNG, JPG, medical CT images, and GIS files) or general documentation files (PDFs, Word, and Excel reports), when these are relevant for metadata management or quality control [4,51,67,74]. Including an option to upload data introduces additional requirements for the platform (eg, data security, standardization of data formats, and robust system performance), as it enables researchers and clinicians to manage and analyze their own information, independent of predefined sources [65]. At the same time, it provides flexibility by allowing researchers to work with original or observational data that aligns with their specific research needs [4], ultimately enhancing the platform’s adaptability and relevance to solve research questions. This combination of characteristics (ie, aggregating data from various sources and allowing users to upload their data) can ensure that the platform maintains a robust and up-to-date collection of data sources, fostering a more collaborative environment that benefits broader research initiatives [2].

Second, data standardization functionalities ensure that data from different sources and in different formats is properly aggregated. Aligned to the goal of GA4GH, the standardization of the data involves harmonizing and unifying data to establish compatible and consistent formats. Overall, standardization is recognized as important, with 34 articles highlighting its relevance [2,4,22,29,31,46,49,51,53,58-60,63,65-68,70-80,82,83,85,87, 88]. However, approaches to data standardization vary by platform. For instance, 9 items only explicitly highlight the importance of standardization (eg, studies by Danahey et al [58], Cappelli et al [76], and Ullah et al [87]), 4 items suggested using libraries and Application Programming Interfaces (APIs), such as HTSJDK is an open source Java library, as a tool to standardize [63,66,71,73], and 3 articles propose using pipelines, such as phenotype-genotype harmonization pipelines, for standardizing data (eg, studies by Crawford et al [59], Das et al [70], and Ochoa et al [49]). The standardization process requires constraints to guide different automated approaches; we mapped the various data models and frameworks that emerged in the literature. These models and frameworks serve as nonfunctional requirements, simplifying efforts toward achieving consistency. However, we observed that despite the need for consistency, 10 articles specifically mentioned using customized data models [22,46,52,60,66,74,76,77,83,89]. As explained by Gill et al [72], a reason may be due to the lack of tools that support the implementation of data standards. Other articles refer to established data models that facilitate standardization, including frameworks such as DataSHaPER [77], PATRIC [89], Genomics Data Commons, Genomic Data Model [53], Variant Call Format [75], and EUROCAST [88]. Additionally, global ontologies [83] play a relevant role in providing consistent vocabularies for standardization. Another method involves converting various genes, proteins, and probe identifiers to a common reference, such as the Ensembl gene identifier [90]. Together, these frameworks, ontologies, and identifier systems underpin efforts to achieve data consistency, enable data sharing, and ensure interoperability across studies and platforms.

Third, data sharing functionalities refer to the ability to distribute, access, and exchange data across various platforms, institutions, or user groups, allowing users to access open-source data while also enabling them to apply for access to proprietary datasets [2,79,87]. These were discussed in 30 items and encompass characteristics, such as collaboration tools, that allow users to work together on specific datasets, with shared access, viewing, and modification rights in certain cases. Collaboration characteristics were mentioned in 25 articles [2,4,29,32,45,48,49,52,53,55,64-67,70,71,74,76-79,82-84,88]. These characteristics facilitate coordinated research efforts and joint analysis among researchers, institutions, or community members, often under controlled permissions. Linked to collaboration, customizable access permissions provide tailored control settings that grant distinct levels of access based on users’ roles or needs within a system. These may include read-only access, full editing rights, or specific access to data, cases, or experiment groups. The flexibility from customizable access permissions helps organizations manage data security, ensure data integrity, and restrict sensitive information as needed while still enabling collaboration (mentioned in 20 articles [2,22,32,49,51-53,55,58,63,65-67,71,74,77,78,83,84,88]). Additionally, the user application for data access is connected to the process of sharing data. User application to data refers to the structured process where users request permission for specific datasets, especially when data sensitivity or security requires controlled access. This process typically involves filling out forms, agreeing to the terms of use, and, in some cases, obtaining approval from a data access committee. This is addressed in 20 articles [2,32,45,47,48,50,51,56,59,66,69,73,76-79,82-84,86].

#### Data Processing and Analysis

The second group among the functional requirements of a well-designed platform of health data management concerns the step of data processing and analysis [4]. This step encompasses 2 essential functionalities (ie, data preprocessing and data analysis methods) aimed at ensuring data quality and deriving meaningful insights (Section S2 in Multimedia Appendix 3). We clustered the functionalities as follows:

First, data preprocessing functionalities allow users to establish standards and parameters to assess the quality, consistency, and suitability of data before computational tasks are undertaken. The functionalities included in data processing involve evaluating data completeness, redundancy, and alignment with research requirements to prepare the data for analysis [35,49,72]. This process aims to ensure that the data are findable (ie, data sharing), accessible (ie, data standardization), interoperable (ie, data standardization), reusable (ie, data acquisition), and fair. In a way, preprocessing the data also entails evaluating its quality. The specific aspect of inspecting data quality was mentioned in 17 articles [35,51,52,57,59,63,66-69, 72-75,81,82,88]. The assessment of the quality can be done by completeness and redundancy (contamination) assessment [72], establishing automated checks that alert users of missing or improperly formatted data [63], evaluating sequencing data with quality control on specific format (ie, bam) alignments, and validation of results from, for example, ChIP-seq, RNA-seq, and ATAC-seq [75]. When the data does not fulfil research requirements, functionalities for data normalization are suggested in 19 articles [4,29,31,35,43,45,47,57,59, 61,63,65,66,68,70,76,77,82,83]. Normalization functionalities aim to make the data consistent by applying various mathematical transformations [66]. Thus, while standardization is typically applied to aggregate data, many studies highlight the importance of verifying data quality and performing normalization before analysis to ensure the data aligns with specific research requirements.

Second, data analysis methods: these functionalities are related to enabling and guiding (pipelines) users to perform different types of analysis (noted in 48 articles [2,4,22,29,31,32,43-50,52-57,59-86,88]. The types of analysis go from sequencing data analysis (such as expressed microRNAs in cancer) and analyzing data from multiple “omics” levels (eg, RNA, protein, and DNA methylation), discussed in 15 articles [2,22,44,49,50,53-55,66,68-70,81,83,85], to statistical analysis that included regressions (discussed in 7 articles [4,31,63,64,70,82,88]). Moreover, 15 articles [4,29,32,44,46,47,49,52,56,62,63,68,71,75,87] mention specialized analysis, like, for instance, pathway analysis; that is, the systematic study of biological pathways, such as metabolic, signaling, or gene regulatory pathways, to understand how specific molecular changes (eg, mutations and gene expression) affect biological processes and diseases. Similarly, network analysis (eg, studies by Pavia et al [35], Raveendran et al [65], Reiff et al [66], and Gill et al [72]) involves examining the relationships and connections between biological entities, such as genes, proteins, metabolites, or pathways, to explore how their interactions shape biological processes and contribute to diseases. To facilitate end users, automated and customizable pipelines are often suggested as key functionalities, that is, a streamlined end-to-end workflow that automatically performs a series of data processing and analysis steps, allowing a standardized approach for analysis, for example, studies by Ullah et al [87], Amer-Yahia et al [83], Post et al [67], and Gill et al [72]. Moreover, the possibility to use command-line option for advanced analysis is remarked in about 15 articles [2,4,22,29,57,59,62,65,69,70,72,75,82,85,87]. Finally, an important aspect highlighted in the data analysis functionality is the inclusion of features that ensure the reproducibility of findings, checking that research findings are consistent, reliable, and can be independently verified. These characteristics were mentioned in 28 articles [2,4,22,29,31,46,48,52,53,55,62-66,70,72,74-78,80-83,86,87]. Together, these data analysis functionalities enable researchers to perform thorough, flexible, and reproducible analyses, enhancing the effectiveness of their insights.

#### Data Visualization and Reporting

The third group of functional requirements for a well-designed platform for health data management focuses on exploring insights through data visualization and producing outputs for reporting (Section S3 in Multimedia Appendix 3). Two of the requirements were identified in the literature.

First, visualizations of data simplify and support the interpretation of complex information, enabling users to identify patterns, trends, and relationships within the data [2,49,91]. Visualization of data was mentioned as a key function in 40 articles [2,4,22,29,31,32,35,43,44,46,47,49-56, 59-66,70,73-76,79-84,87,88]. Data visualization might involve different types of visualizations like networks [56,82,83], scatter plots [35,50,64], genome browsers [55,65,68], heatmaps [54,57,64], pie charts [61,81,88], histograms [35,61,64], and dashboards [63] to present information clearly, easily, engagingly, and understandable.

Second, reporting functionality facilitates the creation of files and documents that effectively communicate the results of the analysis. This ensures that findings can be shared, published, and used for further research or practical applications [2,35,55,82]. The ability to generate reports that provide an overview of the data in different formats and modalities is a well-discussed feature discussed in 22 articles [2,4,29,35,45,46,48,49,51,55,60,64,68,71,73,75,77,80-83,88]. Related to generating reports is the option to download data in different formats, which appears in 31 articles [2,31,32,35,43,44,46,49-53,55,56,59,61,62,64-66,68,70,72-76,80,82,86,87]: Tab-Separated Values, images, text files, and JavaScript Object Notation files. Generation of reports is also connected to knowledge dissemination (mentioned 9 times in articles, such as studies by Reska et al [4], Yukselen et al [55], and Campbell et al [64]), as it facilitates sharing insights with others. To enhance the efficiency of sharing insights, incorporating citation buttons for easy integration into reports is also considered a useful approach [65,87].

### Nonfunctional Requirements

#### Overview

**Figure 3:**
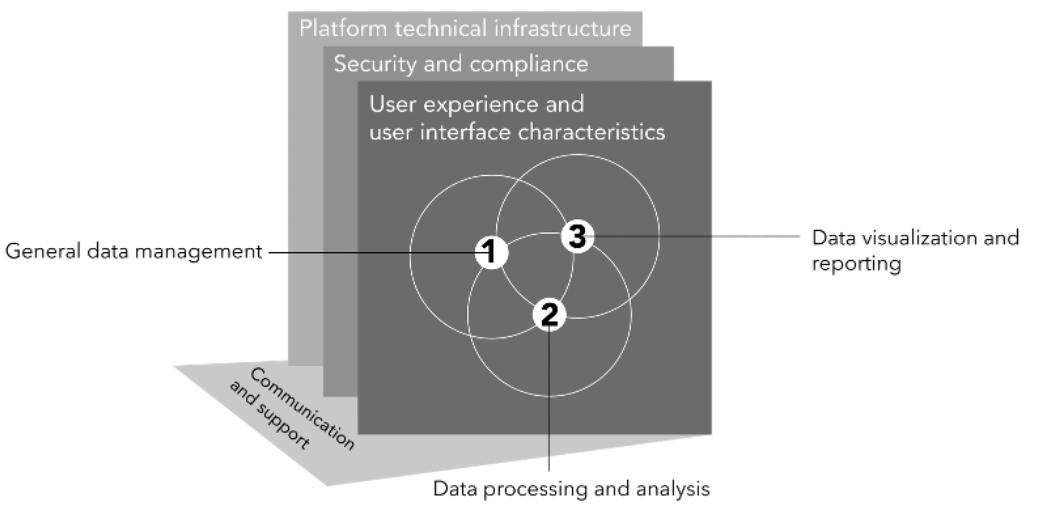
Overview of the different functional and nonfunctional requirements mapped from the literature.

User interactions with a platform are influenced not just by the direct actions that users can perform, but also by the platform’s characteristics (nonfunctional requirements). We identified a total of 20 nonfunctional aspects discussed in the literature, and we clustered such aspects into 4 groups (Multimedia Appendix 4). An overview of the functional and nonfunctional requirements is presented in Figure 3.

#### Communication and Support Requirements

These nonfunctional aspects are a unique intersection of functional and nonfunctional aspects, encompassing both user interactions with the platform and the platform’s autonomous functionalities. The integration of these aspects enables communication between the user and the system while establishing operational constraints. For example, the function of sending reminders a certain amount of time introduces a timeline constraint, a nonfunctional requirement that aims to facilitate compliance. The communication and support functionality comprises 4 primary characteristics. First is *enabling feedback* from internal and external users [85]. This feature was mentioned in 16 articles [2,22,31,44,58,60,61,64,68,72,75,81,83-86], and it refers to information, comments, or data provided to improve, inform decisions to reinforce the improvement of the system.

A second key aspect is to include *documentation about the platform*, for example, to help users understand, navigate, and use a platform effectively. This includes tutorials, user guides, instructional manuals, and technical documentation. Providing users with thorough documentation has been highlighted in 28 articles [2,22,31,32,35,43-46,48,53,55-57,62-64,66,69, 72,74-78,82,83,87], and it serves as a necessary support tool for users to learn about the platform’s characteristics, workflows, and best practices.

A third key aspect is to enable users to set up *notifications*. This feature was mentioned 4 times (eg, studies by Gilbert et al [79] and Osmond et al [71]). Notifications in this context refer to alerts or messages that inform users (such as participants, data managers, or researchers) about new or important updates related to data or research. For instance, when new data becomes available, when participants are eligible to participate in a new study, or when there are updates on the research results. Finally, some articles mentioned that *allowing for communication between patients and clinicians* is also relevant. This feature was mentioned in 2 items [50,79].

#### Platform Technical Infrastructure

For the platform technical infrastructure, we see that a federated approach to data management was mentioned in 8 articles [2,29,53,71,73,74,78,82]. As federated infrastructure enables decentralized data storage and computation, ensuring data privacy by keeping sensitive information local while allowing collaborative analysis across sites, there is a trend over recent years, indicating its growing adoption. Another relevant feature within the architecture was ensuring the scalability and adaptability of the architecture. As noted in 22 articles [4,22,29,31,49,51-54,57,58,62-64,66,67,72,74,80,82,84,85], this feature highlights the need for systems designed to handle future growth, including increased data volume or user demand, to ensure the robustness of applications as needs evolve. Finally, the use of APIs to facilitate the ease of data integration and exchange of information was mentioned in 13 articles [2,22,46,49,51,53,56,60,61,63,65,67,76]. These articles further emphasize the need for scalability and adaptability within the system. The platform architecture category highlights the growing importance of federated data management, scalability, adaptability, and API usage. Federated approaches, as seen in multiple studies, enable decentralized data storage and secure collaborative analysis, reflecting a trend toward broader adoption. The scalability and adaptability of systems, emphasized in numerous articles, ensure that platforms can grow and evolve to meet increasing data and user demands. Additionally, the integration of APIs facilitates seamless data exchange, further supporting the flexibility and expansion of the architecture to accommodate future needs.

#### UX and UI Characteristics

Several key characteristics were highlighted in the Core UI and UX category to improve platform interactivity and UX. For example, the importance of a usable interface was emphasized in 35 articles [4,29,31,35,43,45-50,52,54,55,58,61,62,65-68, 70,71,73,75,76,78-85,87], particularly given that not all users have the technical expertise required to navigate complex programming interfaces for data analysis.

One main feature to make the platforms user-friendly was searching for data, discussed in 36 articles [2,4,22,29,31,32,43-46,49-53,55,58,60,63-67,69-71,73,74,76,77,80,83,84,87,88]. Searching for data refers to the process of locating specific datasets or pieces of information within a more extensive database or data system. This can involve querying databases using various search methods, like entering keywords, applying filters, or selecting specific attributes (eg, accession number, item type, or metadata). Searching allows users to efficiently find relevant data by navigating through records and retrieving data based on specific criteria.

However, other specific characteristics could enhance user interaction with visual data, as highlighted in multiple studies. For example, 10 articles [32,35,43,45,46,60,69,72,83,88] mention the importance of highlighting specific regions, while 11 articles [35,43,50,61,64-66,70,80,84,88] emphasize the usefulness of zooming in on graphs to explore certain genes more in-depth. Additionally, 6 articles [43,46,50,61,84,88] noted the relevance of hover effects. First, highlighting specific regions involves visually distinguishing some aspects of the data, typically using color or other visual cues to make them stand out. This helps users quickly identify and focus on the areas they wish to explore further, such as selecting contigs or data points in a chart or plot. Second, zooming on graphs allows users to dynamically adjust the level of detail displayed, enabling them to magnify specific sections of a graph, map, or plot. Third, hover effects provide users with additional information or functionality when they hover the mouse over a specific element. Together, these characteristics facilitate a more efficient and focused exploration of data, allowing users to analyze trends deeply while maintaining an overview of the broader dataset.

Other relevant characteristics that enhance user interaction with visual data include dropdown menus and drag-and-drop functionality, all of which were discussed across multiple studies. For instance, dropdown menus refer to allowing users to select options or filter data within an interface efficiently, as mentioned in 11 articles [35,45,50,51,58,60,64,66,72,77,84]. This feature is particularly useful for navigating large datasets or switching between different settings without cluttering the UI. Similarly, the drag-and-drop feature (mentioned in 9 articles [31,35,46,55,56,64-66,84]) allows users to easily upload files or rearrange graphical elements by dragging them across the screen. This feature increases the customization of visual data and enhances the interactivity of the platform, making it more intuitive and user-friendly.

Finally, 2 characteristics addressing the accessibility and device compatibility of the platform were having mobile-friendly interfaces (discussed in 8 articles [31,32,44,51,61,65,79,84]) and supporting multiple languages [52].

### Security and Compliance

A total of 4 key characteristics emerged from the literature review in the security and compliance category. The first key feature is the *implementation of privacy-protective measures*. These include methods, such as data deidentification, anonymization, and other data protection measures designed to safeguard sensitive information by obscuring or removing personal identifiers, ensuring compliance with privacy laws and ethical standards. Privacy-protective measures were discussed in 20 articles [2,4,29,32,48,52,54,58,64,65,70,73-75,78,79, 82,84,86,88]. The second feature identified is user registration. User registration is when individuals create an account or register on a platform, often by providing essential information such as a name, email address, and other necessary details. Registered users are usually granted access to more secure or restricted platform areas, ensuring that sensitive data is only available to authorized individuals. This feature (ie, user registration) is noted in 13 articles [2,22,31,52,53,56,59,60,67,71,74,78,84], and it allows platforms to guarantee the security of sensitive data and track activity to maintain accountability while offering more personalized services to the users. Closely related to user registration, the third feature is user authentication. This mechanism continuously verifies users through multifactor authentication and real-time access control processes that monitor and regulate their access to systems or data. Authentication mechanisms were highlighted in 10 articles [52,55,56,58,60,67,70,74,84,86]. The fourth feature is consent management. From the patient’s perspective, this refers to systems that enable individuals to provide, withdraw, or modify consent for how their data are used over time. Notably, 8 articles [4,58,59,63,71,79,82,86] highlighted the importance of consent systems to empower patients to adapt their preferences to changing needs, ensuring respect for autonomy and ethical data use.

## Discussion

### Main Findings

There is a consensus among experts that data-driven and evidence-based health care is possible only if we (1) establish standardized mechanisms for data exchange [92,93]; (2) enable all the actors to provide, access, and analyze the data for specific and regulated purposes [94]; (3) enable the digital transformation in a way that is respectful of the patients’ ownership of the data.

The tension between the need to access and manipulate data for scientific research while respecting the privacy and safety of the patients is at the center of the discussion, often creating fragmentation. The literature reflects an ongoing debate about the features that should be included in health and genomic management platforms. Many of the proposed functionalities aim to facilitate data analysis, making genomic workflows more accessible to stakeholders beyond bioinformaticians (eg, studies by Gill et al [72], Duong et al [95], Almeida and Oliveira [96], and Melles et al [97]). At the same time, the discussion emphasizes the need to strengthen data ownership, privacy, and security (eg, studies by Arneson et al [86], Bonomi et al [62], and Almeida and Oliveira [96]), alongside the development of technical infrastructure that can enable data access, while maintaining privacy, for example, federated vs centralized data governance [98,99]. Together, these elements highlight the underlying sensitivity of genomic data and its importance for advancing precision medicine.

This work attempted to map, organize, and make sense of such a scattered discussion in literature. Our goal was to provide designers and developers with a set of functional and nonfunctional requirements discussed in relation to data management platforms for health care and genomics data that consider their users in the process. The functional and nonfunctional requirements for genomic data management help distinguish operational functionalities from platform qualities. The functional requirements outline the platform’s core features that address the explicit or implicit needs of end users, while the nonfunctional requirements describe the platform’s qualities (eg, accessibility and usability) that ensure these functionalities can be used effectively and appropriately. These mapped requirements can help designers and developers understand diverse user needs and anticipate potential trade-offs before requirements elicitation, ultimately enabling more informed and user-centered design decisions.

The nonfunctional requirements, such as the platform’s technical infrastructure (eg, federated or centralized), security compliance, and UI characteristics, shape its overall functionality. These requirements must align with industry standards and stakeholder needs to ensure effectiveness. By serving as both a framework and a set of constraints, these requirements define the platform’s “possibilities of action” in terms of functioning. Whereas communication and support (eg, manuals, help, and artificial intelligence (AI)–conversational agents) fall under the nonfunctional requirements, as it helps users learn how to use the systems appropriately, it also serves an additional role by compensating for design issues such as missing functionalities, interactive problems, malfunctioning, etc. In this sense, the nonfunctional requirements comprised the infrastructure necessary for integrating the 3 key functional requirements most frequently discussed in the genomic research process.

### Overview of the Functional Requirements

The following functional requirements are relevant to ensure that the platform serves the needs of all key stakeholders.

#### General Data Management

The acquisition, integration, and upload of data from diverse sources is essential. Such platforms must aggregate information from EHRs, laboratory results, open-source databases, and proprietary hospital data to construct robust and comprehensive datasets [4,83]. Some platforms even allow users to upload their own data files (eg, FASTQ, BAM, and VCF) [2,22,45,53]. The diversity of information enhances the research capacity by including data that reflects various real-world scenarios [2,45,52]. However, there are 2 important functionalities that should be considered before the acquisition of the data. First, the standardization of the data is necessary to ensure compatibility across datasets. Implementing predefined templates, controlled vocabularies, and standardized APIs enhances data standardization and can improve data quality and ensure reliability [2,77]. Although data standardization has been crucial in advancing health and genomic research, there remains a lack of consensus on the standards that should be adopted [49]. This issue is critical to address because nonstandardized data are either unusable or require more time and effort to process [49,78]. In this context, although GA4GH represents one of the largest initiatives in the field, it is mentioned in only a few articles. Thus, despite its significance, it remains underrepresented in the review. This could be due to its ongoing nature, the fact that not all standards are universally applicable, and other evolving factors. Therefore, it is essential for the health and genomic data fields to acknowledge more widely the current initiatives (eg, GA4GH, ELIXIR, and GDI), further agree on a unified approach for standardization, and promote it. In fact, the management of sensitive data, such as genomic data, demands adherence to strict regulations and standards. Meeting these standards further depends on the strength of the technical infrastructure. As such, the operationalization and definition of nonfunctional requirements are key to the success of such functionalities. Second, within the acquisition and standardization of the data, it is important to consider the usability and the time of the data application process for stakeholders to access health data [49,78]. To standardize the overall application procedure for consistency might streamline the process, concurrently it could be useful to provide examples or a wizard on the application form to guide users through the process [49,78].

#### Data Processing and Analysis

Genomic research platforms must support diverse types of analysis, including pathway, network, and regression analyses, to address the different research needs [72,83]. Within the analysis functionality, having the ability to run predefined, automated, and customizable pipelines (ie, workflows) is also crucial to ensure efficiency and flexibility in the analysis process to address specific research questions [49,87]. Furthermore, the reproducibility of analytical workflows is essential to ensure that research findings are reliable, independently verifiable, and scientifically credible [72]. These aspects are crucial due to the complexity of the data, where intricate, multistep, and specialized tools are needed [29]. As many analysis workflows resemble tangled spaghetti code rather than standardized, reproducible clinical processes [29], health data management platforms must simplify the analysis process, enabling researchers—including nonexperts—to extract meaningful insights more efficiently. In addition, the integration of AI into the analysis platform can identify patterns in large datasets, recommend the most suitable analytical methods, and predict outcomes based on historical data trends. For instance, pathway and network analyses, frequently used for understanding gene interactions and biological pathways, could be optimized by AI algorithms that adaptively select the most fitting analysis based on specific research questions and dataset characteristics. This capability would not only improve research efficiency but also support more accurate and insightful conclusions in genomics and broader health care research.

#### Visualizing and Reporting Data

This is relevant for making complex data accessible and interpretable. Characteristics such as charts, graphs, and maps help users identify patterns and trends that might be obscured in raw data. Similar to the role of automated and customizable analytical workflows in simplifying data analysis for nonexperts, visualization tools enhance data comprehension and insight extraction [35,56]. In addition, the integration of some nonfunctional requirements, such as highlighting DNA regions, zooming on graphs, and hovering, facilitates detailed exploration of genomic data without overwhelming the user. Allowing for transitions between broad overviews and granular analysis, as well as sensory accessibility, serves to reduce cognitive load and improve data comprehension through dynamic visual cues [100]. When integrated with reporting capabilities, these tools further support the dissemination of analytical findings, enabling more efficient communication of results [82]. In a way, the visualization and reporting characteristics enhance the utility of data management platforms for researchers, facilitating more informed decision-making and collaborative research efforts.

### From Functions to Operationalization

The previous functional requirements mapping can serve as a basis for rethinking any current or future health management platform. However, once the functional requirements are translated into real systems, a second layer of requirements emerges. This second layer contains how functional requirements are operationalized, that is, through nonfunctional requirements. An example of this is the general data management functional requirement. This requirement specifies that the platforms should pull information from EHRs, laboratory systems, open databases, and user-uploaded genomic files, such as FASTQ, BAM, and VCF [1-5,29]. In order to operationalize the functionalities of data management, designers must design an interface that can guide users through complex tasks of visual information retrieval and by implementing search tools that might help users navigate, interact with, and understand large datasets (eg, studies by Mohr et al [74], Cappelli et al [76], Rodchenkov et al [47], and Canakoglu et al [77]). This process could rely on an interface that enables users to examine data at a meta level rather than accessing the underlying individual-level information directly, thereby supporting compliance with current regulatory frameworks [7,11,13]. In practice, this could be achieved through a federated approach in which data remain within institution-controlled nodes [99], and the UX and UI provide access only to metadata or aggregated outputs [2,4]. In this sense, nonfunctional requirements, such as UX and UI design, should not be viewed as merely aesthetic or technical add-ons but as part of an iterative design process that optimizes usability while remaining aligned with additional nontechnical requirements, such as the technical infrastructure and security and compliance protocols.

Platforms that aim to support the needed functions for the users, like multiple analytical methods, customizable workflows, reproducible pipelines, and even AI-assisted insights, should also consider the quality in the operationalization of such functionalities, that is, nonfunctional requirements. We are claiming that the success of future platforms for genomic data exchange is not only in the option of data processing and analysis, but also in the ability of the platform to handle high computational demand in a scalable way. To support this aspect, in literature (eg, studies by Ochoa et al [19], Pang et al [31], Demchak et al [56], and Rodchenkov et al [47]), several strategies were identified for managing high-dimensional datasets. For instance, Sauria et al [57] suggest a message passing interface parallelization process to allow for scalability, while Mohr et al [74] highlight scalability through high-performance computing clusters and cloud services integrated with automated workflows, which distribute analyses across interconnected computer resources. These forms of internal computational scalability also underpin federated infrastructures, where data remain at their source (eg, local hospitals) and computation is distributed across institutions [99]. In a federated approach, each site must possess sufficient local compute power or cloud access to process its own data, enabling multisite analysis without the need for data centralization [99].

Although federated data management was mentioned in only a small number of articles (eg, studies by McLeod et al [2], Wolf et al [73], and Mohr et al [74]), it represents a promising approach for managing health and genomic data. Particularly, since decentralizing data storage and processing ensures that sensitive data remains in one place, reducing privacy concerns while enabling collaborative analysis across multiple sites [2,49,78]. Instead of transferring raw data, machine learning models are trained across decentralized locations, sharing only model updates, such as learned parameters. Similarly, clinicians and researchers are not exposed to raw data; instead, the platform provides structured quality indicators, metadata profiles, and model-ready summaries that allow researchers to understand the suitability of a dataset without directly querying its content. On the one hand, this offers clinicians and researchers an environment that removes the need for programming expertise, enabling broader participation from groups that are traditionally excluded from genomic analytics. On the other hand, the ability for institutions to contribute their datasets through secure, institution-controlled nodes, rather than transferring individual-level genomic data externally, ensures alignment with strict data governance requirements and reduces institutional barriers to adoption. This approach lowers operational friction and makes it feasible for hospitals with diverse policies and technical capabilities to engage in multisite federated studies. More broadly, a federated approach helps preserve data privacy, ensuring compliance with European and American regulations [7,11,13] and the Health Insurance Portability and Accountability Act (HIPAA) Privacy Rule [12]. It also responds to the challenges posed by data sovereignty laws, which restrict the movement of sensitive data across regional or national borders. However, for these projects to succeed, it is essential to enable cross-border collaboration.

### Enabling Collaboration in Federated Infrastructures

For designers and developers aiming to build federated infrastructures, it is crucial to prioritize features that support cross-border collaboration. Such collaboration requires coordination among researchers, legal experts, and technical professionals to develop communities that balance privacy with data accessibility [2,4,32]. Importantly, collaboration in this context hinges not only on technical capability but also on trust and shared expectations. Institutions must have confidence that their data will be handled responsibly and that they will benefit equitably from participation in shared projects, such as developing federated infrastructures. In this regard, although technical infrastructures can facilitate collaboration, the literature also highlights the importance of social and organizational conditions for making such collaboration possible [2,4,32]. For instance, federated infrastructures rely on institutions aligning their data models and colabeling datasets so that shared analyses become feasible [2,29,71]. Conversely, the establishment of a shared infrastructure can itself promote collaboration by providing a common environment, vocabulary, and set of tools through which partners can coordinate their work. This reciprocal relationship, where collaboration is both a prerequisite for and a product of shared infrastructures, extends beyond the definition of functional and nonfunctional requirements. Nevertheless, certain nonfunctional requirements, such as communication features and documentation, can play a fundamental role in the collaboration process. For instance, Holtgrewe et al [48] note that providing tutorials lowers the barrier for new users or institutions to join and contribute to the ecosystem. Similarly, Gill et al [72] highlight that tutorials and protocols help users approach the platform with a shared understanding, while also emphasizing the relevance of responding to user feedback to improve the usability and reciprocity in the collaborations. These examples illustrate how communication and documentation are important to support the collaboration. Yet, despite their importance, we found relatively few examples in literature that explicitly address these requirements. To fill this gap, future genomic data management platforms should incorporate features, such as documentation explaining user roles and responsibilities, real-time notifications that keep participants informed of data access and usage, and feedback mechanisms that allow users to report concerns and suggest improvements. Together, these elements would enhance usability, promote collaboration, and encourage broader adoption.

### Limitations

While this scoping review offers a range of requirements for designers and developers seeking to create an integrated digital ecosystem for genomic and health care data, several limitations should be acknowledged.

First, the review may be affected by publication bias. Studies from regions with well-established digital infrastructure are more likely to be published, whereas experiences from regions with limited infrastructure may be underrepresented. This imbalance can lead to an overemphasis on certain challenges, such as inclusive and robust analysis, while other critical issues, such as financial barriers to implementation, receive less attention. Consequently, the findings may reflect a skewed perspective on what constitutes effective genomic data management, and the applicability of certain solutions may vary depending on local infrastructure and resources.

Second, the search strategy prioritized user-centered design and usability to capture end users’ perspectives on their needs as reported in the literature. This focus enabled the mapping of functional requirements, meaning what users want from these systems, although it offers less insight into methods for implementing such requirements. Although technical papers are referenced, an in-depth discussion of implementation approaches falls outside the scope of this study. Future reviews could target literature on nonfunctional requirements, such as high-performance computing optimization and cloud-native genomic workflows, to better capture the architectural and computational foundations that support scalable genomic analysis. Such work would complement the user-centered perspective presented here by illustrating how various workflows and pipelines are managed at the infrastructure level.

Third, this review focused exclusively on English-language articles, which may have excluded relevant work published in other languages. Additionally, while the inclusion of peer-reviewed articles and gray literature ensures scientific rigor, valuable insights from nonacademic sources, such as industry reports or early-stage studies not yet appearing in peer-reviewed journals, may have been overlooked.

To address these limitations, future stages of this research will adopt a Delphi approach to involve key stakeholders in reviewing and achieving consensus on the findings presented here [101]. This process will be more inclusive, engaging international experts from diverse countries alongside industry professionals and patient advocates, thereby incorporating perspectives that this review could not fully capture.

### Conclusion

The key functional and nonfunctional requirements identified in this work can potentially form the starting point for defining a core set of functionalities (standards) for genomic data management interfaces worldwide. While there is a clear need to define ways to aggregate and protect important personal health data from inappropriate access and use, there is also a tension with the fact that other multiple parties need or want to access such data for different purposes. The complexity of such a sociotechnical system is further increased by the need to enable and maximize the control of the data owners (the patients) over the use of such information. While we leave the definitions of when it is appropriate to access the data, by whom, and for what purposes to the ethics and legal experts, from the human factors and psychological point of view, it is essential to identify what is acceptable, what is considered trustworthy by the stakeholders, and how to communicate to the patients (the owners of the data) and to all other stakeholders their rights, their duties, and their options for action. We suggest that advancements in genomic data management systems should focus on three primary functional capabilities: (1) general data management, including data acquisition, mechanisms for data sharing, and standardization; (2) the preprocessing and analysis of the data; and (3) along with the visualization and reporting of insights derived from that analysis. These functional elements must be supported by nonfunctional requisites, including secure infrastructure, compliance with legal and ethical standards, and structured governance to facilitate controlled and trustworthy data sharing.

We hope that this work will feed a virtuous circle that will rapidly lead to a human-centered design of health data platforms that are able to address different users and their needs, while also addressing key challenges, such as privacy and technical scalability.

## Appendix

Multimedia Appendix 1 PRISMA-ScR checklist.

Multimedia Appendix 2 Criteria used to select the articles.

Multimedia Appendix 3 Functional requirements.

Multimedia Appendix 4 Nonfunctional requirements.

## Abbreviations

AI: artificial intelligence
API: Application Programming Interface
EHR: electronic health record
GA4GH: Global Alliance for Genomics and Health
GDI: Genomic Data Infrastructure
GDPR: General Data Protection Regulation
HIPAA: Health Insurance Portability and Accountability Act
OSF: Open Science Framework
PRISMA-ScR: Preferred Reporting Items for Systematic reviews and Meta-Analyses extension for Scoping Review
TEHDAS: Towards a European Health Data Space
UI: user interface
UX: user experience
