# Supplementary material for: Toward a Common Set of Interface Requirements for Genomic Data Management: Scoping Review
Source: J Med Internet Res. 2026 Apr 27;28:e78405. doi: 10.2196/78405 (PMC13161837; doi:10.2196/78405)
Supplement: Multimedia Appendix 2 [file jmir_v28i1e78405_app2.docx]

**Appendix 2. Criteria used to select the articles**

| *Field* | *Inclusion* | *Exclusion* |
| --- | --- | --- |
| **Population** | This study focuses on articles addressing the perspectives, experiences, and needs of researchers, clinicians, and patients when using health data platforms for clinical studies. | Articles that primarily targeted students or populations not directly involved in research, clinical practice, or patient care were excluded from the analysis. |
| **Publication Type** | Peer-reviewed open-access research articles. | Non-peer-reviewed articles, preprints, conference proceedings, theses, review articles, meta-analyses, commentaries, editorials, and book chapters. |
| **Time** | Articles published between 2014 and 2024 | Articles published before 2014 |
| **Language** | English | Other languages |
| **Comparison** | Not applicable | Not applicable |
| **Outcome** | Empirical and exploratory studies, particularly use cases that describe or evaluate systems designed for health and genomic data management. | Studies that focus solely on the technical aspects of health or genomic data management, such as infrastructure design or coding procedures. |
