# Supplementary material for: Toward a Common Set of Interface Requirements for Genomic Data Management: Scoping Review
Source: J Med Internet Res. 2026 Apr 27;28:e78405. doi: 10.2196/78405 (PMC13161837; doi:10.2196/78405)
Supplement: Multimedia Appendix 3 [file jmir_v28i1e78405_app3.docx]

## S1. Functional requirement - General data management


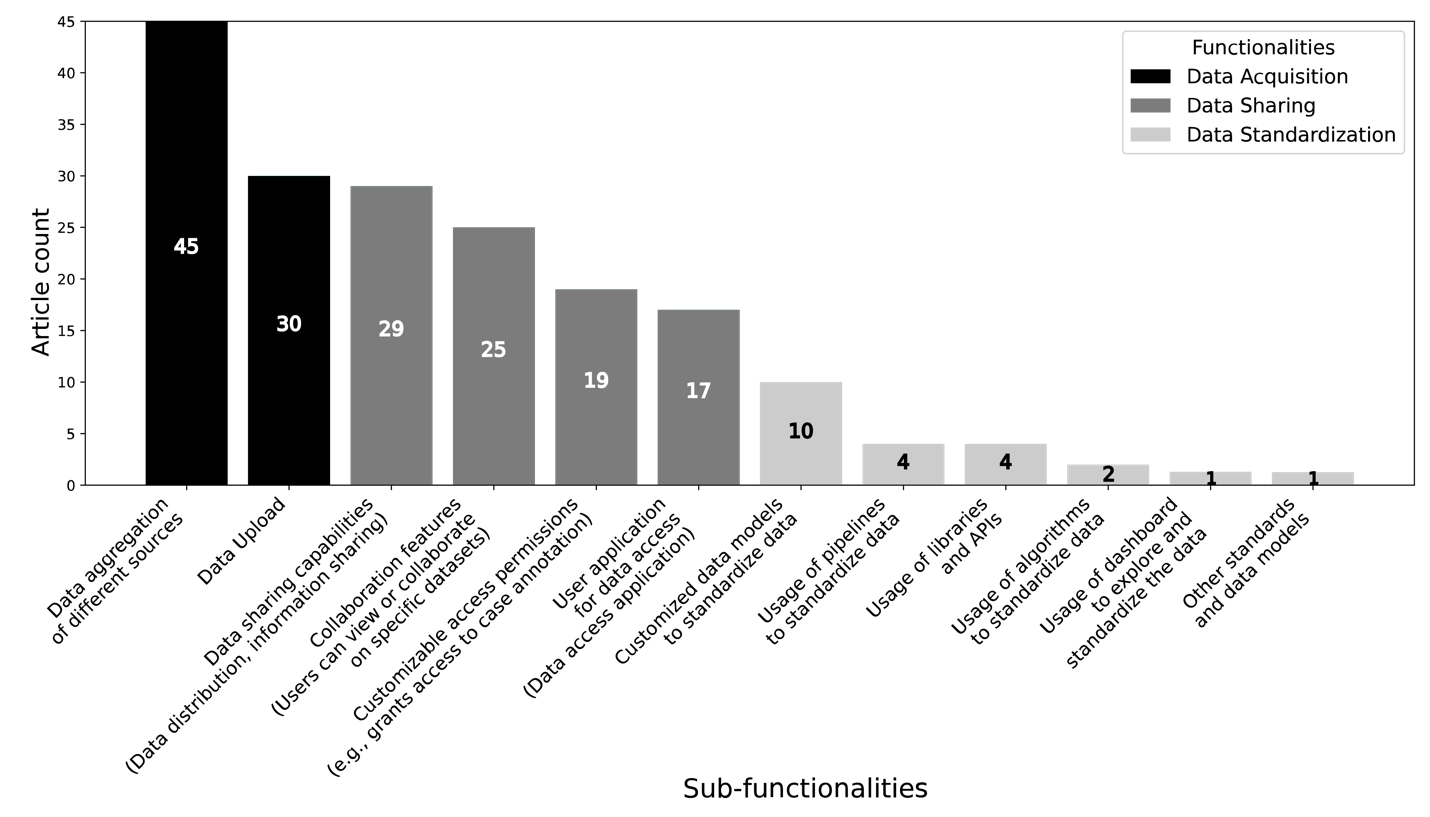


| **Functional requirement - General data management** | **N. of articles that mention the functionalities** | **Articles that mention the functionalities (%)** | |
| --- | --- | --- | --- |
| **DATA ACQUISITION** |  | |  |
| 1. Data aggregation of different sources | 45 | | 84.9% |
| 1. Data upload | 30 | | 56.50% |
| **DATA STANDARDIZATION** |  | |  |
| 1. Usage of libraries and APIs | 4 | | 7.55% |
| 1. Usage of pipelines to standardize data | 4 | | 5.66% |
| 1. Usage of algorithms to standardize data | 2 | | 3.77% |
| 1. Usage of dashboard to explore and standardize the data | 1 | | 1.89% |
| 1. Customized data models | 10 | | 18.87% |
| 1. Others* (DataSHaPER harmonization model 2. Integrate PATRIC and RCBPR, Genomics Data Commons (GDC), Ensembl, Variant Call Format, Adopting nomenclature standards 3. EUROCAST, Global ontologies) | 1 | | 1.89% |
| **DATA SHARING** |  | |  |
| 1. Data sharing capabilities (Data distribution, information sharing) | 29 | | 54.71% |
| 1. Collaboration features (Users can view or collaborate on specific datasets) | 25 | | 47.16% |
| 1. Customizable access permissions (e.g., grants access to case annotation, experiment groups, have a read-only access or full edit rights) | 19 | | 35.84% |
| 1. User application for data access (Data access application) | 17 | | 32.07% |

## S2. Functional requirement - Data processing and analysis


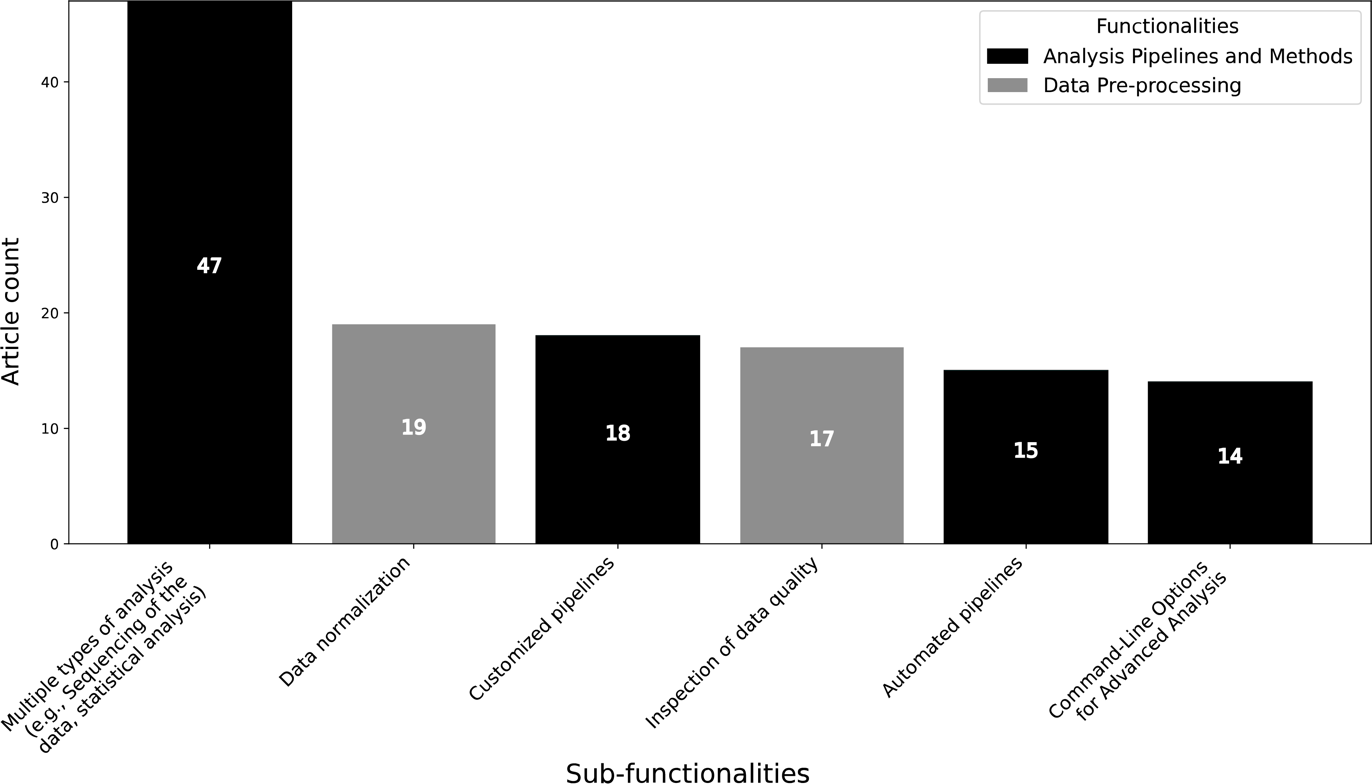


| **Functional requirement - Data processing and analysis** | **N. of articles that mention the functionalities** | **Articles that mention the functionalities (%)** |
| --- | --- | --- |
| **DATA PRE-PROCESSING** |  |  |
| 1. Support the inspection of data quality | 17 | 32.07% |
| 1. Allow for the normalization of data | 19 | 35.84% |
| **DATA ANALYSIS METHODS** |  |  |
| 1. Ability to conduct multiple types of analysis (e.g., sequencing data, statistical analysis) | 47 | 88.67% |
| 1. Command-Line Options for Advanced Analysis | 14 | 26.41% |
| 1. Reproducibility of findings | 27 | 50.94% |
| 1. Automated pipelines (i.e., running automatically the workflows) | 15 | 28.30% |
| 1. Customized pipelines (i.e., fine-tuning the workflows) | 18 | 33.96% |

## S3. Functional requirement - Data processing and analysis

***
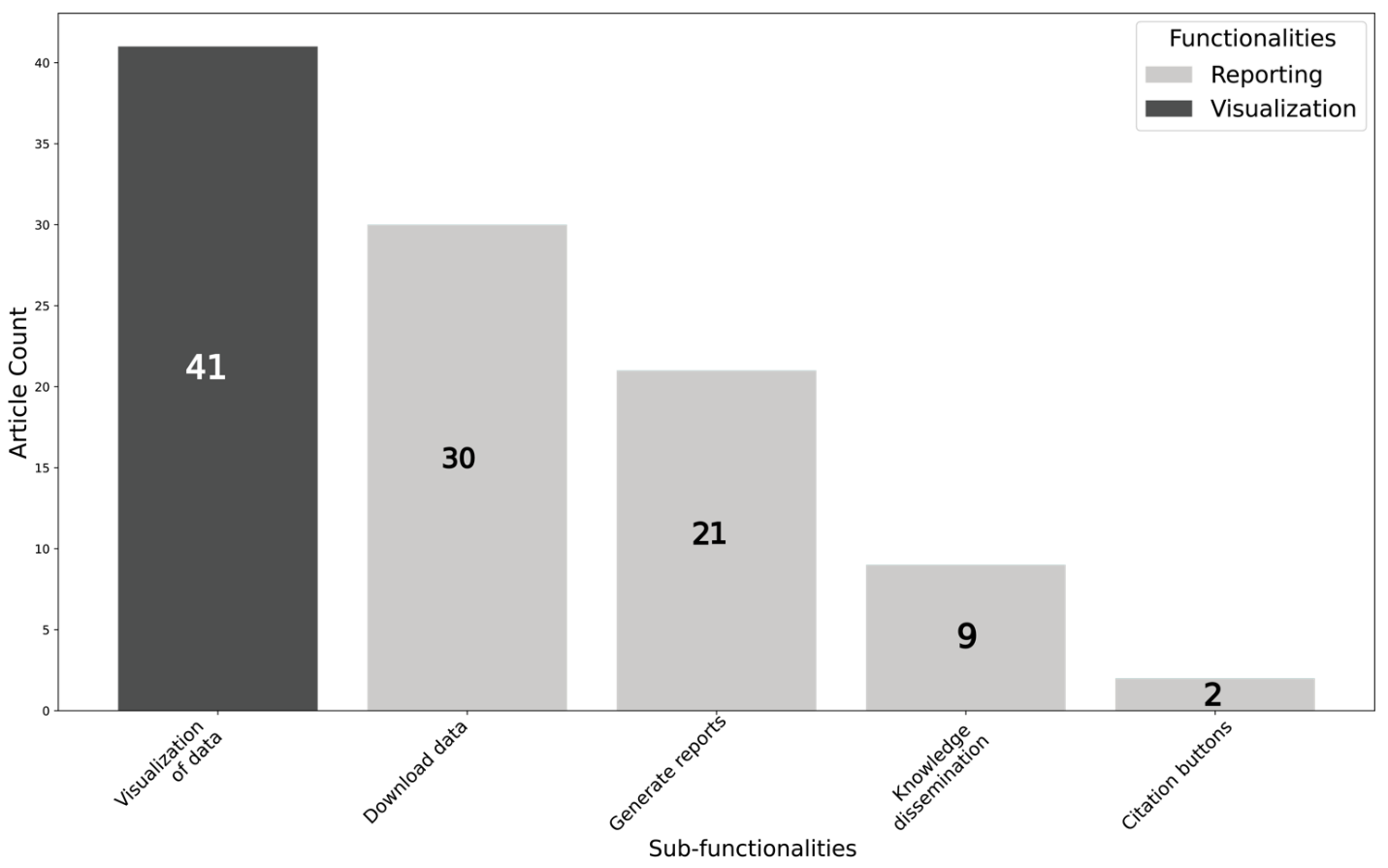
***

| **Functionalities- Data visualization and reporting** | Examples | **N. of articles that mention the functionalities** | **Articles that mention the functionalities (%)** |
| --- | --- | --- | --- |
| **VISUALIZATION** |  |  |  |
| 1. Visualization of data |  | 41 | 77.36% |
|  | Networks | 6 | 11.32% |
|  | Scatter plots | 3 | 5.66% |
|  | Genome browser | 3 | 5.66% |
|  | Heatmaps | 6 | 11.32% |
|  | Pie charts | 3 | 5.66% |
|  | Histograms | 3 | 5.66% |
|  | Dashboards | 1 | 1.89% |
| **REPORTING** |  |  |  |
| 1. Generate reports |  | 21 | 39.62% |
| 1. Download data |  | 30 | 56.6% |
| 1. Knowledge dissemination |  | 9 | 16.98% |
| 1. Citation buttons |  | 2 | 3.77% |
