# Supplementary material for: Toward a Common Set of Interface Requirements for Genomic Data Management: Scoping Review
Source: J Med Internet Res. 2026 Apr 27;28:e78405. doi: 10.2196/78405 (PMC13161837; doi:10.2196/78405)
Supplement: Multimedia Appendix 4 [file jmir_v28i1e78405_app4.docx]

# **Appendix 4. Non-functional requirements**

| **Non-functional requirements** | **N. of article that mentions the aspect** | **Articles that mention the aspect** |
| --- | --- | --- |
| **COMMUNICATION AND SUPPORT** |  |  |
| 1. Feedback from users | 17 | 32.07% |
| 1. Documentation | 27 | 50.94% |
| 1. Notifications | 4 | 7.54% |
| 1. Communication between patients and clinicians | 2 | 3.77% |
| **PLATFORM ARCHITECTURE** |  |  |
| 1. Federated infrastructure | 8 | 15.09% |
| 1. Scalability and adaptability | 21 | 39.62% |
| 1. Application Programming Interfaces (APIs) | 13 | 24.53% |
| **UX/UI** |  |  |
| 1. Usable/user-friendly interface | 35 | 66.04% |
| 1. Searching for data | 36 | 67.92% |
| 1. Highlighting specific regions | 9 | 16.98% |
| 1. Zooming in on graphs | 10 | 18.87% |
| 1. Hover effects | 5 | 9.43% |
| 1. Dropdown menus | 10 | 18.87% |
| 1. Drag-and-drop functionality | 8 | 15.09% |
| 1. Mobile-friendly interfaces | 7 | 13.21% |
| 1. Supporting multiple languages | 1 | 1.89% |
| **SECURITY AND COMPLIANCE** |  |  |
| 1. Privacy-protective measures | 20 | 37.73% |
| 1. User registration | 8 | 15.09% |
| 1. User authentication | 10 | 18.86% |
| 1. Consent management | 11 | 20.75% |
